# Successful cultivation of edible fungi on textile waste offers a new avenue for bioremediation and potential food production

**DOI:** 10.1038/s41598-024-61680-5

**Published:** 2024-05-20

**Authors:** Liberty Hazelgrove, Suzy Clare Moody

**Affiliations:** School of Life Sciences, Pharmacy and Chemistry, Kingston University, Kingston Upon Thames, KT1 2EE UK

**Keywords:** Fungi, Environmental biotechnology, Environmental impact

## Abstract

Textile waste contains both natural fibres such as cotton and bamboo viscose, and synthetic fibres such as elastane and polyester. As a complex mixture, textiles present a challenging pollution issue as breakdown in landfill results in microplastics entering water and soil environments, and incineration results in particulate air pollution. Here the use of edible fungi as bioremediation agents of waste textiles is described for the first time. Three species of filamentous fungi were shown to colonise and grow on mixed fibre textile waste (underpants made from 28% cotton: 68% bamboo viscose: 4% elastane). All three fungi were able to metabolise the common textile dye Reactive Black 5 to some extent. The metabolome was captured to elucidate the dye remediation pathway utilized and to characterise the volatiles released during bioremediation with a view to assessing the safety profile of this process for future industrial applications. The results suggest that edible fungi may be cultivated on textiles, and that some interesting and useful compounds may be produced in the process. This has great biotechnological potential. No mushrooms were produced in this study, suggesting that further work will be needed to optimise conditions for crop production from waste textiles.

## Introduction

Textile waste is a large and growing environmental pollution issue that is largely ignored by the wealthy countries driving fast fashion trends and consumerist lifestyles^1^. The majority of textile production, processing and disposal occurs in low- and middle-income countries (LMICs)^2^ who are typically poorly regulated or resourced to cope with the scale of the waste production or develop sustainable routes to improving the fashion industry’s environmental toll^3^. Additionally, textiles are usually a mixture of natural and petroleum-derived synthetic fibres which present a complex recycling challenge. Current understanding of the eco-toxicological impacts of textile waste have been summarized elsewhere^4^. The current interest in circularity and sustainability have given rise to new biotechnological processes utilizing textile waste in a range of applications^5–7^. These developments are largely researched by industrialized, wealthy nations and involve expensive equipment, environmentally damaging processing or are marketed under patent. While the burden of environmental pollution from the textile industry is borne by LMICs, few remediation or recycling options have been designed specifically to be adaptable to these resource poor settings. The project described here was designed to begin to address the global inequalities associated with textile waste and offer a low-cost, accessible platform for developing fungal bioremediation as a sustainable means of reducing the environmental cost of fashion.

Filamentous fungi are designed to explore their environment through production of a hyphal network, capturing nutrient resources and colonizing them while they utilize them for growth. Wood decay fungi are a specialist group of filamentous fungi well-adapted, in some cases, for complete mineralization of complex polyphenolic substrates such as lignocellulose. They belong to a polyphyletic group which specializes in utilizing dead plant materials in the forest floor ecosystem, playing a key role in the carbon cycle. Extracellular enzymatic action of low-specificity peroxidases and laccases enables utilization of woody resources that are rich in carbon, while providing limited nitrogen which is typically bound to the polyphenols present^8^. It is these attributes that suggest these fungi as potential bioremediation agents of textile waste.

The concept of purposeful introduction of exogenous bioremediating microbes into a contaminated closed-system environment is known as ‘bioaugmentation’ and as such utilizes seeding of microbes into the pollutant to initiate bioremediation in situ (reviewed in^9^, and an experimental example utilizing filamentous fungi in^10^). The filamentous nature of wood decay fungi and their ability to explore their environment is highly beneficial to enable growth and colonization of solid textiles. Waste textiles present a complex and heterogeneous substrate with the presence of natural fibres such as cotton, highly processed natural fibres such as viscose, synthetic petroleum-derived fibres such as polyester and elastane, combined with a range of dyes which often contain azo groups (–N=N–) and represent a pollution issue in their own right^11^. Previous works have documented fungal growth on natural fibres and nylon (a petroleum-derived synthetic) but these have had short timeframes and utilized lab conditions^12,13^. Likewise, several studies have addressed bioremediation of azo dyes such as Reactive Black 5 (RB5) used in this study but typically in the context of wastewater^11,14^ rather than remediation post-use in the textile industry. The chemical structures of synthetic textile polymers such as elastane and nylon and the RB5 dye share aromatic groups and carbon abundance in common with lignocellulose, raising the possibility of bioremediation of heterogeneous textile waste by wood decay fungi^15^.

The premise of this work was that utilization of textile waste as a substrate by filamentous fungi was a potential bioremediation tool that could be adapted to resource poor settings where the impact of environmental damage is most felt. Further, in LMICs nutrition is often sub-optimal and if edible fungi could be safely cultivated on textile waste, this would offer a significant source of protein as a beneficial side-effect of remediation. This paper describes the rationale for choosing certain trial fungi, their growth in agar-free microcosms containing semi-synthetic textiles dyed with RB5 (black pants), and the volatile and substrate-level metabolome produced as a result. It is the first project to address the question of whether we could eat our own pants courtesy of fungal bioremediation and mushroom production.

## Results

### Fungal selection

The selection criteria used for choosing which fungi to trial were: the mushroom produced by the fungus should be an acceptable food source across the globe; laboratory cultivation should have been demonstrated in the literature; there should be a publicly available genome; strains should be obtainable from an established culture collection. Based on these criteria, four species of fungi were identified and taken forward for further investigation (Table 1).

**Table 1:** The trial species with the edible mushroom they produce listed.

Group	Species	Strain	Mushroom produced
White-rot basidiomycete	*Pleurotus ostreatus*	DSM 11191	Oyster mushroom
	*Pleurotus eryngii*	DSM 9619	King oyster mushroom
	*Lentinula edodes*	DSM 3565	Shiitake mushroom
Litter-decomposing basidiomycete	*Agaricus bisporus*	DSM 1003	Button mushroom

### Bioinformatics

**Figure 1:**
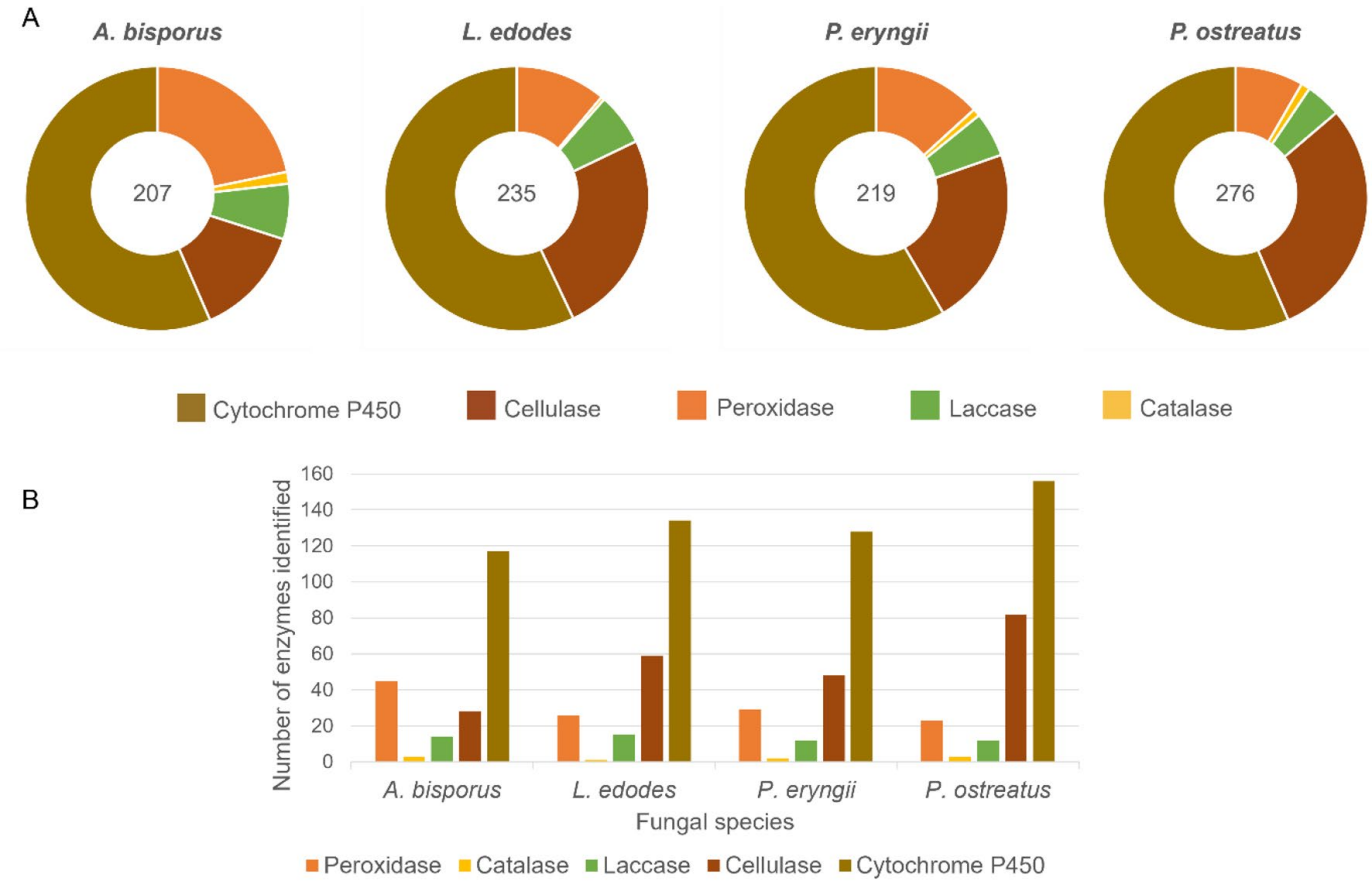
Comparison of ligninolytic enzymes thought to be important for textile degradation across the four species of fungi. (**a**), the proportion of the different enzyme classes identified in each genome with the number in the centre of the pie charts representing the total number of ligninolytic enzymes identified for that species of fungus. (**b**) Gives a comparison of the numerical breakdown of each enzyme class in each fungus. All data were taken from JGI MycoCosm (Mycocosm (doe.gov).

An overview of the enzymatic capacity of the four fungal candidates was conducted using the Joint Genomes Initiative website^16^ for bioinformatic analysis of genomes and predicted proteins. This was limited to 5 key enzymes groups that have been associated with lignocellulosic breakdown, and are therefore hypothesized to have potential for degradation of semi-synthetic textiles. The results of this genome comparison are shown in Fig. 1.

Laccases and peroxidases have previously been suggested as important candidates for breakdown of the RB5 dye^17,18^. While all species had very similar numbers of laccases (all in the range 11–15), the peroxidase complement showed greater variance between species with *A. bisporus* having almost double the number of peroxidases of *P. ostreatus*. In contrast, *P. ostreatus* had by far the most cellulases with 25% more than the next nearest, *L. edodes*. As cellulases were considered critical for growth on and metabolism of the natural components of the textile (which make up 96% of available substrate), a further analysis of the cellulase complement of the trial fungi was conducted, the results of which are shown in Table 2. *P. ostreatus* contained many more cellulases associated specifically with breakdown of cellulose rather than other carbohydrate moieties, compared to the other fungi. In particular, it was shown to have 31 enzymes predicted to be capable of endohydrolysis of (1- > 4)-beta-D-glycosidic bonds in cellulose (EC:3.2.1.4), and 13 enzymes for breaking the same bonds at the non-reducing ends of cellulose chains (EC:3.2.1.91). It was noted that *P. eryngii* was considerably different in complement to *P. ostreatus*, despite belonging to the same genus. *P. eryngii* had high numbers of enzymes capable of hydrolysing the bond between two different types of carbohydrate or between a carbohydrate and a non-carbohydrate moiety (glycosyl hydrolase (GH) families 5 and 7). For *L. edodes* and *A. bisporus* one of the most abundant enzyme activities was glucan 1,3-beta glucosidase (EC:3.2.1.58). (This was also present in both *Pleurotus* species but represented a lesser proportion of the cellulase complement.) The significance or advantage these enzymes may offer to textile bioremediation is as yet unknown as the cellulose available in the substrate is highly processed and likely to be physically and biochemically different from that encountered in their respective ecological niches.

**Table 2:** The cellulase complement of the trial fungi.

Cellulase type	Entry number	Number of genes identified on JGI Mycocosm database			
		*P. eryngii*	*P. ostreatus*	*L. edodes*	*A. bisporus*
Cellulase	EC:3.2.1.4	1	31	17	1
Cellulase (glycosyl hydrolase family 5)	PF00150	11	7	4	8
Glucan 1,3-beta glucosidase	EC:3.2.1.58	9	12	16	13
Mannan endo-1,4-beta-mannosidase	EC:3.2.1.78	0	2	4	0
Cellulose 1,4-beta-cellobiosidase (non-reducing end)	EC:3.2.1.91	0	13	4	0
Glucan endo-1,3-alpha-glucosidase	EC:3.2.1.59	3	6	8	3
Glucan endo-1,3-beta-D-glucosidase	EC:3.2.1.39	0	2	0	0
Glycosyl hydrolase family 45	PF02015	2	2	0	0
Xyloglucan-specific endo-beta-1,4-glucanase	EC:3.2.1.151	0	1	4	0
Xyloglucan-specific endo-processive beta-1,4-glucanase	EC:3.2.1.155	0	2	1	0
Glycosyl hydrolase family 12	PF01670	2	1	0	2
Glycosyl hydrolase family 26	PF02156	0	0	1	0
Glycosyl hydrolase family 7	PF00840	20	3	0	1
		Total 48	Total 82	Total 59	Total 28

All cellulases identified by annotation in the JGI genome database for each fungus were included, and the EC number in the annotation was used to identify function where possible.

Based on these results, there was no obvious candidate more likely to succeed in colonizing and potentially degrading textiles so all four fungi were taken forward for laboratory trials.

### Imaging and growth

The initial stage of setting up the textile microcosm involved culture of the fungus onto a wood chip that was then used to inoculate the textile. *A. bisporus* grew well on malt extract agar but would not extensively colonize either beech or pine wood chips. When the poorly colonized wood chips were trialed on textiles, *A. bisporus* did not grow out (given a time period of 2 months). This may be due to a different ecology underpinning *A. bisporus* as it is a leaf-litter specialist rather than a woody lignocellulose degrader per se. This fungus was not used in any further experiments. No such issues were encountered with *P. ostreatus, P. eryngii* or *L. edodes* which all colonized beech wood chips successfully, and subsequently grew out on textiles in the microcosms.

**Figure 2:**
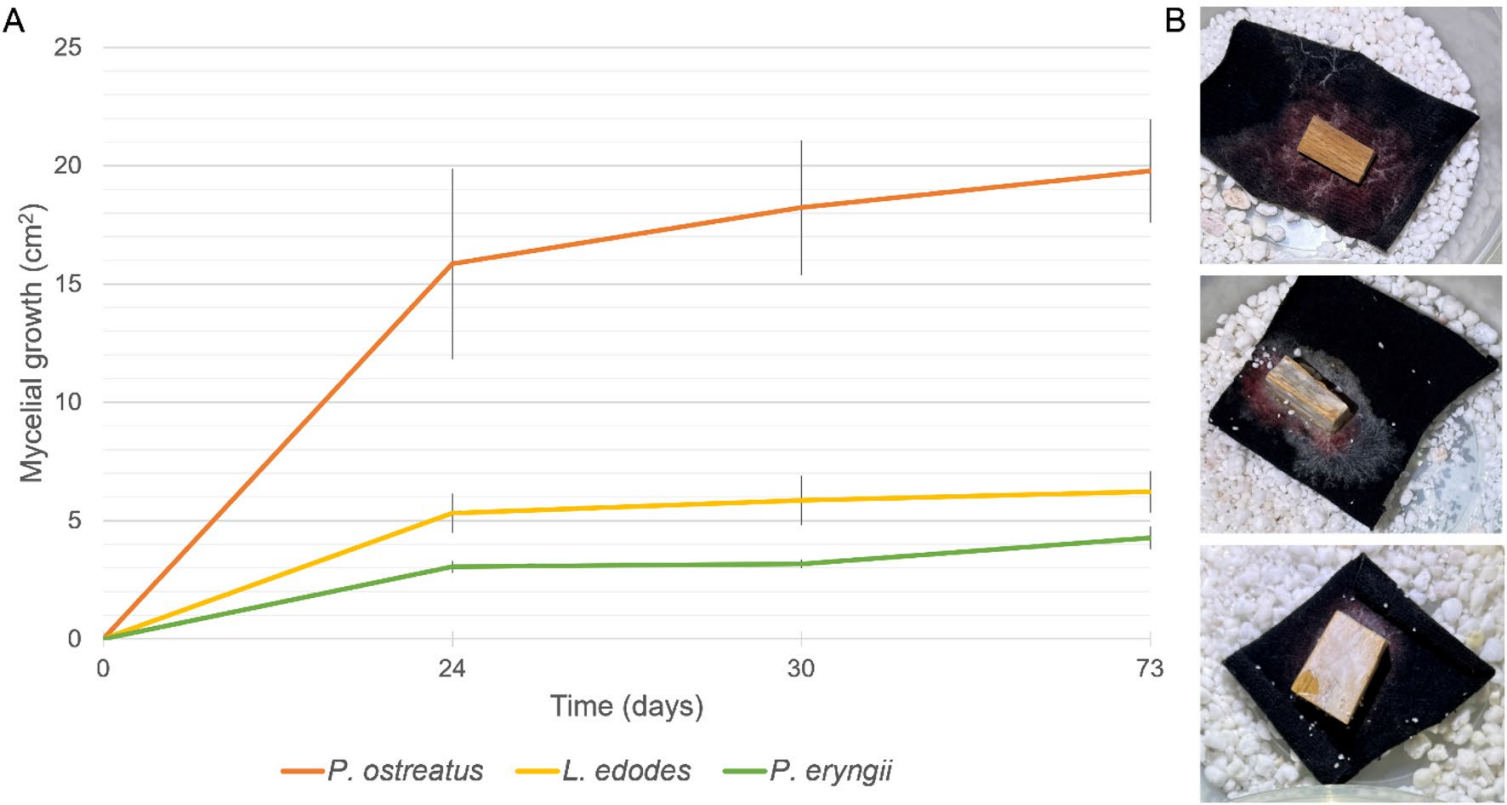
(**A**) Growth curve of textile colonization from day 0 to day 73 for *P. ostreatus*, *P.eryngii* and *L. edodes*. The images used for the growth curve were analysed in ImageJ to generate the numerical data. Each line represents the mean of three triplicate microcosms, with standard error bars shown. (**B**) image of the microcosms at 73 days incubation with *P. ostreatus* at the top, *L. edodes* in the middle and *P. eryngii* at the bottom.

Photographic images of the microcosms were used to document outgrowth of fungal hyphae from the wood chip over a period of 10 weeks. Fungal growth was not equal, with *P. ostreatus* showing greater coverage than either *P. eryngii* or *L. edodes* (Fig. 2). It should be noted that any of these could be an underestimate of growth as growth through or under the textile would not be included. Neither *P. eryngii* or *L. edodes* reached the margin of the textile by the 73 day time point. The triplicates of *L. edodes* demonstrated some variability with two microcosms having quite poor growth and one (pictured in Fig. 2B) managing more extensive coverage.

### Scanning electron microscopy (SEM) imaging

**Figure 3:**
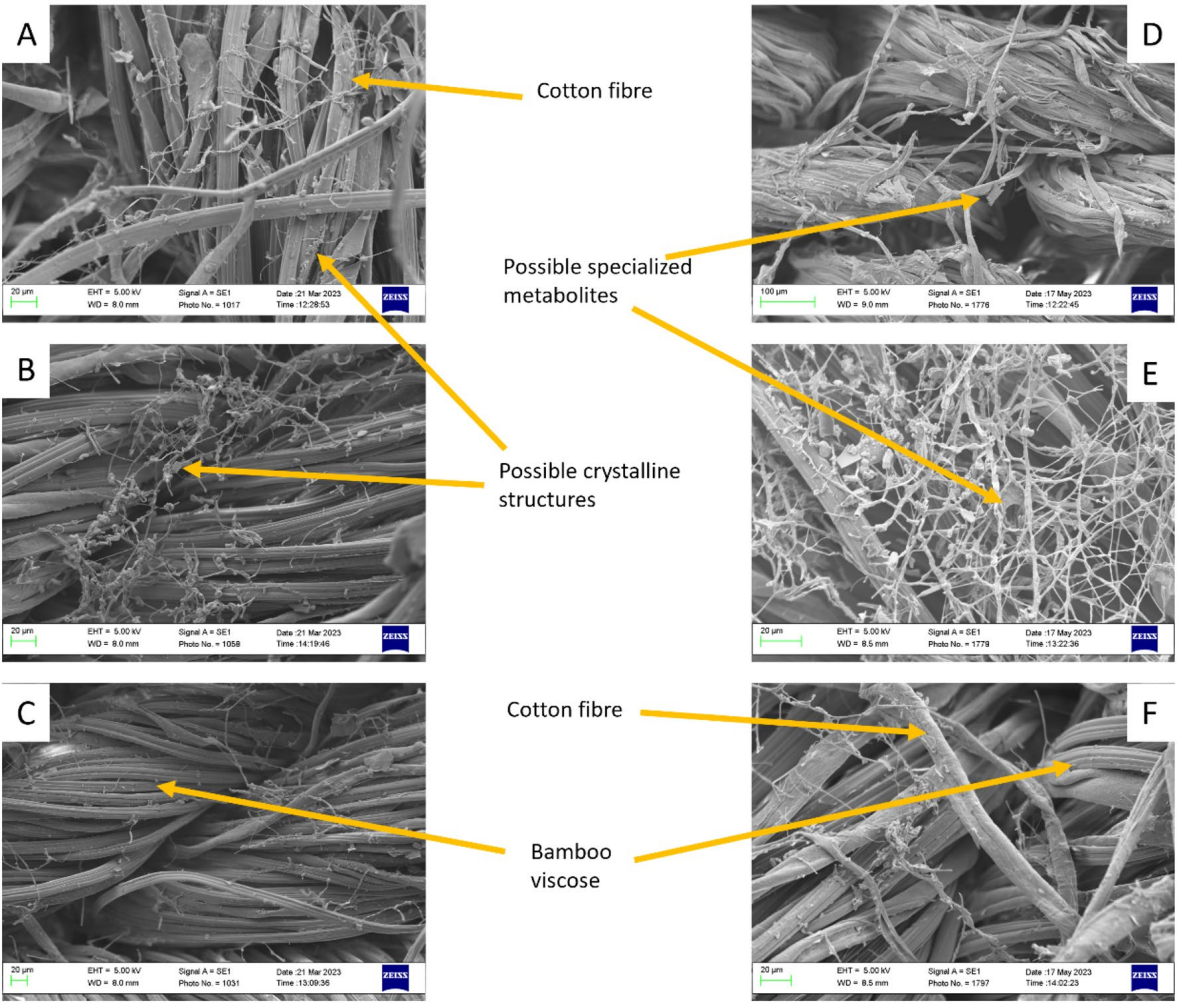
A representative sample of SEM images taken at 3 months (left panel) and 5 months (right panel). (**A** and **D**) show *P. ostreatus*, (**B** and **E**) show *L. edodes*, and (**C** and **F**) show *P. eryngii*.

Sections of colonized textiles were sampled at 3 months and 5 months and imaged by SEM (Fig. 3). The images presented here are a representative sample to highlight some key findings.

The cotton and bamboo viscose fibres are clearly visible in most of the images. These can be distinguished as bamboo viscose shows extrusion lines along the length of the threads, while cotton threads are of a similar width but appear flatter, sometimes slightly curled at the edges but lacking extrusion marks. The elastane fibres were not visualized, but as previously reported, they are typically only visible at the margins of the textile^15^. All three fungal species appeared to grow on both cotton and bamboo viscose suggesting that the high level of processing involved in viscose production does not reduce bioavailability of the cellulose to the fungus. The hyphal growth, seen as an interconnected mycelial network particularly in *L. edodes*, appeared normal compared with previous works^15,19,20^.

The presence of possible crystals (3 months) and specialized metabolites (5 months) was noted in the *P. ostreatus* and *L. edodes* samples. Putative metabolites have been noted in others’ images and show as a sheet-like structure on SEM imaging^21^. Crystalline structures are a common feature seen in SEM images of fungi. Those visualized in *L. edodes* here bear some resemblance to the characteristic shape of those seen and identified previously as whewellite (calcium oxalate monohydrate)^22,23^, while those visualized in *P. ostreatus* microcosms appear less regular so their identity, composition and possible role remains unknown.

### Dye extraction and quantification

**Figure 4:**
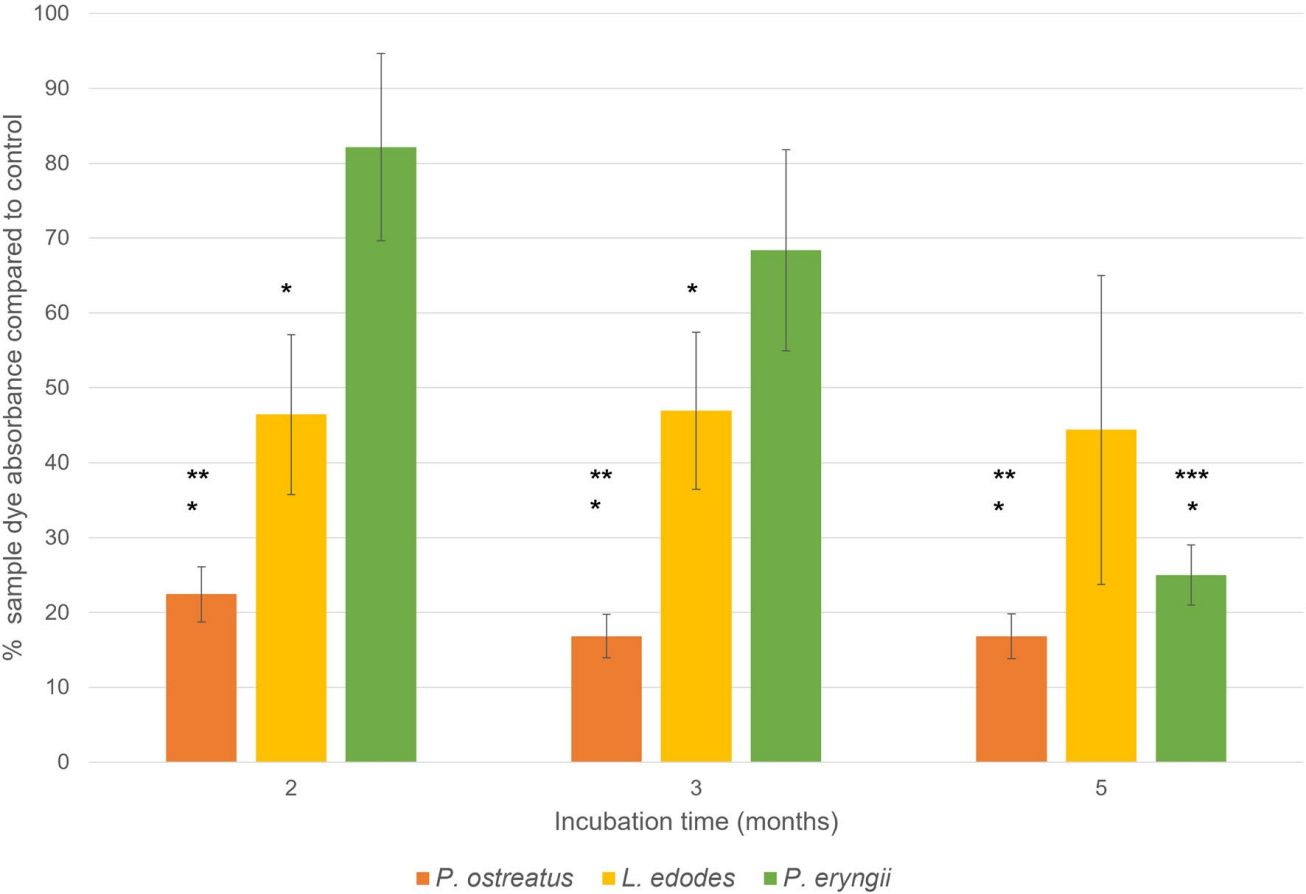
The percentage dye loss from textiles at 2, 3 and 5 months incubation with either *P. ostreatus*, *L. edodes* or *P. eryngii* compared to the control. Each column represents the mean of three triplicate microcosms. The stars indicate samples that were significantly different. (*samples significantly different to the control, as determined by one-sample T-test, **P. ostreatus significantly different to *L. edodes* and *P. eryngii*, as determined by a Two-Factor ANOVA, ***P. eryngii at 5 months significantly different to 2 months and 3 months, as determined by a Two-Factor ANOVA).

As revealed in the imaging, visible dye loss causing a bleaching effect to give a red coloured spot on the textile was noted in some of the microcosms. A sodium hydroxide extraction was used to quantify this dye loss from the textiles over time and the results are shown in Fig. 4.

In concordance with the visible changes to the textiles over time, the extraction quantified this dye loss confirming that *P. ostreatus* metabolized significantly more of the dye than either of the other species and that this was manifest from the earliest time point (*P. ostreatus* vs. *L. edodes* *P* = 0.020, *P. ostreatus* vs. *P. eryngii* *P* =  < 0.001). *P. eryngii* showed a trend for dye loss over time (20 weeks vs. 7 weeks *P* = 0.005, 20 weeks vs. 12 weeks *P* = 0.035), and the change by 5 months was significantly different to the control (*P* = 0.003) although to a lesser extent than *P. ostreatus*. *L. edodes* dye content was significantly different to the control over the first two time points (2 months *P* = 0.038, 3 months *P* = 0.037), but not at 5 months incubation. This may be due to the greater variability seen in *L. edodes* microcosms mentioned earlier (evidenced by the larger error bars seen on the graph) which is reflected in the power value (below 80%) for this one-sample t-test, suggesting this may not have been strong enough to pick up a small effect.

### Identification of dye breakdown products

The presence of red colouration in the textiles is suggestive of breakdown of RB5, and this was noted in the microcosms of all three fungi at 5 months incubation. To identify any known RB5 breakdown products, the trial textiles were extracted in ethyl acetate and the resultant mixture analyzed by GCMS. The results were compared with published examples of fungal RB5 degradation. One of the known breakdown products of ascomycete RB5 bioremediation is 2,4-di-tert-butylphenol^24^, which was detected in all replicates of all three fungal species. The full list of compounds detected in each replicate is in Supplementary Information Table S1.

### Metabolomics

Two methods of metabolite capture were used: substrate level capture utilizing a capture resin applied to the fabric, and volatile capture facilitated using a fibre suspended directly above the open microcosm. These complementary techniques were used to give an overview of the metabolites released into the fabric as a result of fungal enzymatic activity, and those given off as volatiles during textile bioremediation. This is essential information for assessing the safety of fungal bioremediation and identifying compounds released into the immediate environment as a result. Only those compounds found in at least two replicates are reported (see Supplementary information Table S2 for substrate level capture results and Table S3 for volatile capture results).

### Substrate-level metabolite capture

2,4-di-tert-butylphenol, the RB5 breakdown product specifically identified above, was also detected in the substrate level capture in the microcosms of all three fungi, at all time points, suggesting that RB5 bioremediation begins early on in the textile colonization and was underway before visual discolouration occurred. A sulphur-containing compound, specifically propanoic acid, 3-mercapto-, dodecyl ester, was found in all three fungal microcosms at 2 months, and also in the 3 month samples for both *Pleurotus* species. The most likely source of sulphur in these microcosms is the RB5 dye. Propanoic acid, 3,3′-thiobis-, didodecyl ester and bis(2-ethylhexyl) phthalate, both identified in the *L. edodes* substrate-level metabolome, bear significant similarity to breakdown intermediates identified in^25^ as part of a community-mediated degradation pathway to complete mineralization of the azo dye acid blue 113.

Antioxidant compounds were a feature of the substrate level metabolite capture with 2-tert-butylhydroquinone detected at 3 months incubation in *L. edodes* and *P. ostreatus* microcosms; benzophenone isolated from *L. edodes* at 3 months; 1-octadecene isolated at 3 from *L. edodes* and 3 and 5 months from *P. ostreatus*. *P. eryngii* did not seem to use the same antioxidant strategies for growth on textiles as no compounds with this property were consistently identified. Bioactive compounds were identified in all metabolomes. In *P. ostreatus* this included hexacosane, methyl stearate, hexadecenoic acid methyl ester and bromoacetic acid hexadecyl ester; in *P. eryngii*, octacosane; and in *L. edodes*, hexacosane, tetracosane and 11-Octadecenoic acid, methyl ester. The significance of these compounds is unknown. The list of compounds identified from each fungus is included in Supplementary Information Table S2.

### Volatile metabolite capture

Toluene, a petrochemical derivative, was seen in *L. edodes* and *P. ostreatus* volatile metabolomes which was not surprising given the presence petroleum-derived elastane in the trial textiles. Degradation of toluene via the action of specific fungal peroxidases has been previously reported^26^, so the presence of toluene in the volatile ‘effluent’ may be temporally limited. Long chain alkanes were also a feature across the range of microcosms. Several of the compounds identified have hazard warnings associated with them, which is important information when considering scale up and industrial safety of textile bioremediation. The full list of volatiles captured is included in Supplementary Information Table S3.

## Discussion

Current estimates suggest that less than 1% of textiles are recycled to similar quality, around 12% are recycled to lower value applications and over 70% are incinerated or sent to landfill. Underpants are a paradigm for mixed synthetic and natural fibre textiles which present a complex recycling challenge. While elastane ensures that underwear fits properly and is functional during consumer use, it also means the garment is harder to recycle and has a much longer biodegradation time compared to textiles made from entirely natural fibres^27^. The need for innovation in this field is clear and should stand within the context of low-tech, affordable developments suitable for global application. This feasibility study assessed the possibility of growing edible fungi on mixed-fibre textiles as a means of bioremediating and valorizing textile waste. The study contributes new knowledge to the field of bioremediation as edible fungi have not previously been shown to grow on textiles, and this is the first demonstration of dye remediation directly from textiles.

The data presented here suggest that although enzyme complements are similar between many different edible fungi, the ability to grow these on textiles does vary quite significantly. Together, the growth curve data (Fig. 2A), the microcosm images (Fig. 2B) and the SEM imaging (Fig. 3) suggest that *L. edodes* may not achieve the textile coverage that *P. ostreatus* does, but it may have denser growth covering a smaller area. Both *P. eryngii* and *P. ostreatus* appear to have grown more sparsely, but *P. ostreatus* achieved a higher area of coverage with a quicker growth rate. Sadly, the most commonly consumed mushroom, *A. bisporus*, did not perform well and failed to colonise the textiles despite repeated attempts and variations.

There have been several suggested RB5 breakdown pathways using different fungi, predominantly using liquid culture to assess the potential of using fungi as bioremediation agents for dye-contaminated water. None of the breakdown products suggested for pathways in *Aspergillus*^28^ or the basidiomycetous wood decay specialist *Trametes*^18^ were identified in the samples in this study. Somewhat surprisingly, the RB5 breakdown metabolites identified by^24^ using the ascomycete *Trichoderma atroviride* did provide a match (Table S1). Their study identified metabolites including 1,2,4-trimethylbenzene, 2,4-ditertbutylphenol, and benzoic acid-TMS which they suggest are predominantly the result of laccase activity. The proposed pathway is initiated with cleavage of the azo bond in RB5 to give a naphthalene triol, the oxygenated ring of which is then cleaved to give 2-(2-carboxy-ethyl)-6-hydroxy-benzoic acid. This in turn undergoes decarboxylation and methylation to give 2,4-ditertbutylphenol. Given the absence of the complex aromatic amines intermediates associated with the *Aspergillus* and *Trametes* pathways, it seems likely that the bioremediation of RB5 by *P. ostreatus*, *P. eryngii* and *L. edodes* proceeds via an oxidative laccase-driven pathway similar to that reported for *Trichoderma*. As aromatic amines are known for their genotoxic effects, the probability that the fungi in this trial do not produce them during RB5 breakdown is a benefit to their suitability for textile bioremediation. Sulphur-containing compounds have been identified as intermediates in fungal RB5 breakdown^18,24,28^ but the sulphur compound identified in this study ‘propanoic acid, 3-mercapto-, dodecyl ester’ has not been identified during RB5 breakdown previously. This may be indicative of a novel breakdown process being utilized by the trial fungi in this study.

This is the first time a potential RB5 breakdown pathway has been reported for *P. ostreatus*, *P. eryngii* and *L. edodes*, and demonstrates the ability of these fungi to bioremediate the dye rather than merely decolourizing it in situ. Further work is needed to characterise the laccase(s) responsible for mediating this xenobiotic pathway in each species. The bioinformatic analysis undertaken in this project (Fig. 1) identified 15 laccases in *L. edodes* and 12 in each *Pleurotus* species. For greater understanding and potential biotechnological engineering of this process, it would be useful to know which laccases are involved and which other enzymes may contribute e.g. members of the cytochrome P450 family which are known for their xenobiotic breakdown and highly enriched in all three trial fungi.

White rot wood decay fungi are known to produce hydroxyl radicals during the wood decay process and it was anticipated that the same degradation mechanisms would be deployed by the fungi in this trial, thereby also generating free radicals in the extracellular space. Antioxidants are considered to be required by the fungi as free radical scavengers to protect the fungal membrane and wall during resource utilization^29^. 2-tert-butylhydroquinone was detected at 3 months incubation in *L. edodes* and *P. ostreatus* microcosms. Hydroquinone is known to be produced by many organisms, including *Agaricus hondensis*^30^ and it is a known precursor for biosynthesis of 2-tert-butylhydroquinone, produced by the action of hydrogen peroxide, catalases and peroxidases^31^ which the trial fungi have in abundance. Production here is speculated to be utilizing the antioxidant properties of 2-tert-butylhydroquinone to protect the lipid component of the fungal cell wall from peroxidation while the extracellular enzymatic processes associated with textile bioremediation occur. Other antioxidant compounds were also identified in the *L. edodes* and *P. ostreatus* metabolomes, suggesting that this is a key fungal protection mechanism during resource utilization. This has been shown elsewhere and suggested that the production of antioxidants by fungi may offer an avenue of pharmaceutical applications^32^. This would be a further opportunity to explore in valorizing textile waste through fungal bioremediation.

While this work demonstrated the ease with which edible fungi can be cultured on semi-synthetic textiles, the conditions in this study were not conducive to mushroom production. Further work is anticipated to succeed in finding environmental conditions suitable for fruiting bodies to be produced. Comparison with other studies reveals *L. edodes, P. ostreatus* and *P. eryngii* have been grown on various agricultural waste sources to investigate the possibility of growing oyster and shiitake mushrooms^33–35^. Unsurprisngly, *P. ostreatus* and *P. eryngii* rapidly colonise wheat straw, which is typically used as a substrate for mushroom cultivation due its accessible structure rich in nitrogen, phosphorus, potassium, magnesium, and iron^35,36^. High growth rates for *P. ostreatus* have also been observed on beech wood shavings, barley and oats straw, corn cobs and rice bark, whereas *P. eryngii* has been observed to have high growth rates on corn cobs, olive pulp and coffee residue^35^. The production of mushrooms may be attributed to the high carbon/nitrogen and cellulose/lignin ratios of these substrates^35^ which would suggest textile waste as a suitable substrate for mushroom production.

The nutritional composition of mushrooms is predominantly as a protein source, which is not anticipated to alter with the change of substrate. Mushrooms produced on this substrate would need full investigation for carryover of harmful compounds or microplastics if considering them for human consumption. This study has demonstrated the removal of dyes from the fabric and their probable breakdown. As the colour of the hyphae remains white, simple adsorption is very unlikely, however, carryover of azo compounds and other elastane or dye breakdown products into the fruiting body is a possibility. There is also the potential for the elastane to be partially broken down and for microplastics to be incorporated into the flesh of the fruiting body. While neither scenario can be ruled out at this stage, both compound and microplastic analyses are easy to conduct once fruiting bodies are produced. The presence of either would preclude use of the mushrooms for consumption but it may be that hyphae grown on textile waste can be used in a range of other biotechnological applications.

This highly novel study has demonstrated that edible fungi *L. edodes*, *P. ostreatus* and *P. eryngii* will all grow on semi-synthetic textiles and are capable of azo dye bioremediation to some extent (Fig. 4). The production of antioxidants by *P. ostreatus* and *L. edodes,* demonstrated on textile waste for the first time, is an interesting avenue to explore in terms of high-value compounds that could be extracted from these microcosms. While consumption of mushrooms grown on waste textiles has not yet been achieved, this is a promising field to explore further.

## Materials and methods

### Fungal strains

Actively growing cultures of *Pleurotus ostreatus* DSM11191, *Pleurotus eryngii* DSM9619, *Lentinula edodes* DSM3565 and *Agaricus bisporus* DSM1003 were obtained from DSMZ (Deutsche Sammlung von Mikroorganismen und Zellkulturen GmbH) culture collection, summarized in Table 1. All fungi were cultured on 2% malt extract agar prior to starting the experiments and incubated at 25 °C in the dark.

### Textiles

The textiles supplied by Bamboo Clothing Ltd (UK) were underpants comprised of 68% bamboo viscose, 28% organic cotton, 4% elastane and dyed with Reactive Black 5 (RB5). The trial textiles were washed in a domestic washing machine using a non-biological washing powder 5 times at 30 °C with 1000 rpm spin cycle, and line dried outside in between each wash. This removed the finishing products widely used in the fashion industry and simulated some ‘wear and tear’ prior to use in the lab.

### Microcosm set up and maintenance

The method used for this was reported in detail in^15^ and is described briefly here. Chips (5 mm × 20 mm × 2 mm) of Scots Pine (*Pinus sylvestris*) and European Beech (*Fagus sylvatica*) were placed on 0.5% malt extract agar, along with a 5 mm × 5 mm chunk of fungal colonized agar from an established 7 day old 2% malt extract plate (fungi sampled from the growing edge of the colony). *P. ostreatus, P eryngii* and *L. edodes* were cultured on beech, *A. bisporus* culture was attempted on pine but this was unsuccessful. The chips were incubated for 3 weeks in the dark at 25 °C to allow the fungi to colonise the wood chips. 250 ml sterile polypropylene pots had a layer of sterile perlite added, followed by 5 ml sterile water, then the textile squares cut to approximately 50 mm × 50 mm placed on top. The fungal colonized wood blocks were removed from the agar, excess agar and mycelium were scraped off, and the wood chip placed on the textile. The pot was sealed, with airholes added for oxygen availability (covered with breathable tape) and incubated in the dark at 25 °C for up to 8 months, with regular checks and addition of 5 ml sterile water if the perlite was dry. 10 replicates were set up for each condition to allow destructive sampling during the project.

### Bioinformatics

The data for the bioinformatic analyses were obtained from the Joint Genomes Initiative^16^ website pages for each fungus (https://mycocosm.jgi.doe.gov/Pleery1/Pleery1.home.html), (https://mycocosm.jgi.doe.gov/PleosDSM11191_1/PleosDSM11191_1.home.html), (https://mycocosm.jgi.doe.gov/Led_CS584_1/Led_CS584_1.home.html) and (https://mycocosm.jgi.doe.gov/Agabi_varbisH97_2/Agabi_varbisH97_2.home.html) (accessed June 2023). The standard search function of annotations was used to identify the enzymes by name and all proteins returned by the search were included in the analysis. The deeper analysis of cellulases was conducted using the individual enzyme names (e.g. glycosyl hydrolase) as search terms in the genome annotations, and EC numbers were used to assign putative function.

### Imaging

The microcosms were imaged using an iPhone 13 mini, operating system iOS 16.5. The images were saved as JPEG files. ImageJ 1.53t was used to analyse and quantify the growth on the photographic images. The straight segmented freehand line option to 'draw' across the width of the pot was utilised, with the scale set to 12 cm (the diameter of the pot), followed by outlining the mycelial growth. All statistical analysis was performed using IBM SPSS software, version 28. The fungal growth data were not normally distributed, so a non-parametric Kruskall–Wallis test was used, followed by a Bonferroni multiple correction test.

The trial textiles were imaged using scanning electron microscopy (SEM, Zeiss Evo 50, Zeiss, Germany) at 2 months post-inoculation (to capture initial colonization of the textile), 3 months and 5 months. (*P. eryngii* was not imaged at 2 months at it was not sufficiently grown out of the wood chip at that point.) 1 cm × 1 cm samples of the textiles were removed from the microcosms and affixed to titanium stubs. A sputter coater was used to coat one side with gold/palladium, with the machine set to 2 kV for 2 min at 20 mA, with Argon pumped into the chamber and maintained at a pressure of 10–7 mbar/PA. The beam of the SEM was set to 5 kV. Images were taken at magnifications ranging from × 138 to × 2176, and a representative sample is included here.

### Metabolomics and gas chromatography mass spectrometry (GCMS) analysis

The controls used in both metabolomics experiments were uncolonized textiles and beech wood. The compounds identified in the controls were removed from the sample compound lists and not included in further analyses.

### Substrate level

This was conducted at 2, 3 and 5 months incubation. 1 g of autoclaved Amberlite XAD16N resin was added to the microcosm and incubated overnight. The resin was gently scraped off each microcosm using a sterile scalpel into separate Falcon 15 ml conical centrifuge tubes. 10 ml 50:50 ethyl acetate: methanol was added to each tube and placed on a rotating platform at 120 rpm for 1 h to extract metabolites before being dried completely in a centrifugal evaporator using the –OH setting at room temperature. The dried samples were stored in a − 80 °C freezer until ready for analysis. 90 μl ethyl acetate was used to reconstitute the sample in glass Chromacol vials immediately prior to GCMS analysis on an Agilent 7890A GC system with 5975C inert XLEI/CI MSD with triple axis detector (Agilent, USA). The reconstituted samples were spiked with 10 μl of 0.001% dodecane in ethyl acetate to act as an internal control for sensitivity and detection.

The GCMS protocol was: from a starting point of 80 °C for 2 min, temperature was ramped to 280 °C, at a rate of 10 °C increase per minute with a hold time of 4 min for a run time of 26 min with a final ramp to 320 °C at a rate of 20 °C increase per minute with a hold time of 1 min. The total run time was 29 min.

### Volatile capture

This was conducted at 3 and 5 months incubation. Microcosms were opened for 1 h in a sealed bell jar with a StableFlex™ 2 cm SPME fiber used to capture volatiles. The samples were immediately loaded by direct injection for GCMS analysis on an Agilent 7890A GC system with 5975C inert XLEI/CI MSD with triple axis detector and the 7683 Injector (Agilent, USA). The protocol for volatile samples was: from a starting point of 80 °C, temperature was ramped to 280 °C, at a rate of 5 °C increase per minute for a run time of 40 min with a final ramp to 300 °C at a rate of 20 °C increase per minute with a hold time of 4 min. The total run time was 45 min.

### GCMS data analysis

A qualitative metabolomics analysis was undertaken to identify the major metabolites present in the microcosms. Total Ion Chromatograms (TICs) were generated using Chemstation, and the NIST 2.0 database (National Institute of Standards and Technology, Gaithersburg, USA) used for identification of matches. For the liquid samples, the whole TIC was analysed and all peaks with greater abundance than the dodecane spike were included in the analysis. A quality cutoff point of 60 on the NIST score was used. For the volatile capture data, peak analysis was conducted between 6 and 30 min retention time, selecting peaks with 50,000 abundance or greater, and with a NIST value of 60 or more. The initial 6 min was dominated by large peaks of toluene and xylene which were also present in the control samples.

The following databases were used to identify functions or health hazards associated identified compounds (accessed June 2023): MetaCyc Metabolic Pathway Database version 20^37^; PhytoHub version 1.4^38^; the Yeast Metabolome Database version 2.0^39^; the Metabolomics Workbench (https://www.metabolomicsworkbench.org/search/index.php); and PubChem^40^ for identification of potential hazards.

#### Dye extraction and quantification

This methodology was developed by^41^ and adapted for this work by^15^. In brief, 1 cm × 1 cm textile was taken from the microcosm and placed in 1 ml of 1.5% sodium hydroxide, vortexed for 30 s and incubated on a heat block at 95 °C for three minutes. The sample was diluted 1 in 4 with 1.5% sodium hydroxide, before dye absorbance was measured at 597 nm with a Helios Epsilon Spectrophotometer (Thermo Scientific, USA). Textile with no fungi growing on it was used as a control. For dye quantification comparing the different species, data was normally distributed, so a two-factor ANOVA analysis was performed, followed by a Sidak multiple correction test. To compare dye loss to the control, one sample t-tests were performed, followed by a power analysis.

#### Identification of dye breakdown products

To identify dye breakdown products present in the microcosm at 5 month incubation, 1 cm^2^ textile sample were taken from the microcosms (and one from control uncolonized textile) and placed in 2 ml ethyl acetate for 1 h at room temperature. The textile was then removed and the liquid filtered through a 0.2 μm filter before being vacuum dried to a final volume of 500 μl for GCMS analysis.

### Supplementary Information

Supplementary Tables.

## Data Availability

All .RAW files and GCMS data are available freely and directly from the Kingston University Data Repository https://researchdata.kingston.ac.uk/190/.
