# Supplementary material for: Successful cultivation of edible fungi on textile waste offers a new avenue for bioremediation and potential food production
Source: Sci Rep. 2024 May 20;14:11510. doi: 10.1038/s41598-024-61680-5 (PMC11106075; doi:10.1038/s41598-024-61680-5)
Supplement: Supplementary file 1 — Supplementary Tables. [file 41598_2024_61680_MOESM1_ESM.pdf]

Supplementary information Hazelgrove and Moody, 2024

Table S1. The retention time, NIST ID and NIST score (quality) of the compounds identified by GCMS in all 5 month samples of textile following extraction in ethyl acetate. PO = *Pleurotus ostreatus*, PE = *P. eryngii*, LE = *Lentinula edodes*.

Sample Number	Sample Description	Retention Time (min)	NIST ID	Quality
91	PO7	Scan 271 (5.637 min)	1,2,3-Propanetriol, 1-acetate	78
		Scan 1017 (9.906 min)	Tetradecane	96
		Scan 1280 (11.411 min)	2,4-Di-tert-butylphenol	97
		Scan 1455 (12.412 min)	Hexadecane	98
		Scan 1642 (13.482 min)	2-Propenoic acid, pentadecyl ester	91
		Scan 1981 (15.422 min)	Phenol, 4-(1-methyl-1-phenylethyl)-	97
		Scan 2512 (18.460 min)	Phenol, 4,4'-(1-methylethylidene)bis-	98
		Scan 2200 (16.675 min)	Eicosane	96
		Scan 2659 (19.302 min)	4,4'-(1,3-Dimethylbutylidene)bisphenol	87
		Scan 2773 (19.954 min)	9-Octadecenamide, (Z)	93
92	PO2	Scan 272 (5.643 min)	1,2,3-Propanetriol, 1-acetate	83
		Scan 1017 (9.906 min)	Tetradecane	96
		Scan 1280 (11.411 min)	2,4-Di-tert-butylphenol	97
		Scan 1454 (12.407 min)	Hexadecane	97
		Scan 1645 (13.500 min)	Cyclododecane	93
		Scan 1844 (14.638 min)	Octadecane	97
		Scan 1980 (15.416 min)	Phenol, 4-(1-methyl-1-phenylethyl)-	97
		Scan 2151 (16.395 min)	Phenol, 4-(1-methyl-1-phenylethyl)-, acetate	97
93	PO8	Scan 271 (5.637 min)	1,2,3-Propanetriol, 1-acetate	83
		Scan 810 (8.722 min)	5-Acetoxymethyl-2-furaldehyde	96

		Scan 1022 (9.935 min)	Tetradecane	91
		Scan 1279 (11.405 min)	2,4-Di-tert-butylphenol	97
		Scan 1453 (12.401 min)	Hexadecane	97
		Scan 1643 (13.488 min)	Cyclododecane	92
		Scan 1981 (15.422 min)	Phenol, 4-(1-methyl-1-phenylethyl)-	97
		Scan 2150 (16.389 min)	Phenol, 4-(1-methyl-1-phenylethyl)-, acetate	97
		Scan 2198 (16.664 min)	Eicosane	98
		Scan 2511 (18.455 min)	Phenol, 4,4'-(1-methylethylidene)bis	98
94	PE5	Scan 271 (5.637 min)	1,2,3-Propanetriol, 1-acetate	63
		Scan 1279 (11.405 min)	2,4-Di-tert-butylphenol	97
		Scan 1454 (12.407 min)	Hexadecane	97
		Scan 1644 (13.494 min)	Cyclododecane	92
		Scan 1844 (14.638 min)	Octadecane	98
		Scan 1980 (15.416 min)	Phenol, 4-(1-methyl-1-phenylethyl)-	97
		Scan 2511 (18.455 min)	Phenol, 4,4'-(1-methylethylidene)bis-	98
		Scan 2777 (19.977 min)	9-Octadecenamide, (Z)-	96
		Scan 2819 (20.217 min)	Tetracosane	99
95	PE4	Scan 269 (5.626 min)	1,2,3-Propanetriol, 1-acetate	83
		Scan 1019 (9.918 min)	Tetradecane	96
		Scan 1279 (11.405 min)	2,4-Di-tert-butylphenol	97
		Scan 1453 (12.401 min)	Hexadecane	97
		Scan 1642 (13.482 min)	2-Propenoic acid, tridecyl ester	91
		Scan 1643 (13.488 min)	Cyclododecane	92
		Scan 1982 (15.428 min)	Phenol, 4-(1-methyl-1-phenylethyl)-	97

		Scan 2149 (16.383 min)	Phenol, 4-(1-methyl-1-phenylethyl)-, acetate	98
		Scan 2199 (16.670 min)	Eicosane	97
		Scan 2510 (18.449 min)	Phenol, 4,4'-(1-methylethylidene)bis	98
		Scan 2774 (19.960 min)	9-Octadecenamide, (Z)-	99
96	PE2	Scan 271 (5.638 min)	1,2,3-Propanetriol, 1-acetate	78
		Scan 1020 (9.923 min)	Tetradecane	96
		Scan 1279 (11.405 min)	2,4-Di-tert-butylphenol	97
		Scan 1455 (12.412 min)	Hexadecane	96
		Scan 1646 (13.505 min)	Cyclododecane	92
		Scan 1845 (14.644 min)	Octadecane	98
		Scan 1977 (15.399 min)	Phenol, 4-(1-methyl-1-phenylethyl)-	97
		Scan 2199 (16.669 min)	Eicosane	97
		Scan 2508 (18.438 min)	Phenol, 4,4'-(1-methylethylidene)bis-	98
		Scan 2522 (18.518 min)	Docosane	98
		Scan 2780 (19.994 min)	9-Octadecenamide, (Z)-	91
		Scan 2821 (20.229 min)	Tetracosane	98
97	LE7	Scan 273 (5.649 min)	1,2,3-Propanetriol, 1-acetate	64
		Scan 1017 (9.906 min)	Tetradecane	97
		Scan 1278 (11.400 min)	2,4-Di-tert-butylphenol	97
		Scan 1456 (12.418 min)	Hexadecane	96
		Scan 1643 (13.488 min)	Dodecyl acrylate	91
		Scan 1846 (14.650 min)	Octadecane	97
		Scan 1978 (15.405 min)	Phenol, 4-(1-methyl-1-phenylethyl)-	97
		Scan 2148 (16.378 min)	Phenol, 4-(1-methyl-1-phenylethyl)-, acetate	97

		Scan 2198 (16.664 min)	Eicosane	97
		Scan 2511 (18.455 min)	Phenol, 4,4'-(1-methylethylidene)bis-	98
98	LE5	Scan 274 (5.655 min)	1,2,3-Propanetriol, 1-acetate	83
		Scan 812 (8.733 min)	5-Acetoxymethyl-2-furaldehyde	97
		Scan 1016 (9.900 min)	Tetradecane	96
		Scan 1279 (11.405 min)	2,4-Di-tert-butylphenol	97
		Scan 1454 (12.407 min)	Hexadecane	98
		Scan 1643 (13.488 min)	Dodecyl acrylate	91
		Scan 1841 (14.621 min)	Octadecane	96
		Scan 1980 (15.416 min)	Phenol, 4-(1-methyl-1-phenylethyl)-	97
		Scan 2149 (16.383 min)	Phenol, 4-(1-methyl-1-phenylethyl)-, acetate	97
		Scan 2199 (16.669 min)	Eicosane	98
		Scan 2511 (18.455 min)	Phenol, 4,4'-(1-methylethylidene)bis	98
99	LE1	Scan 271 (5.638 min)	1,2,3-Propanetriol, 1-acetate	78
		Scan 1020 (9.923 min)	Tetradecane	96
		Scan 1276 (11.388 min)	2,4-Di-tert-butylphenol	96
		Scan 1454 (12.407 min)	Hexadecane	96
		Scan 1643 (13.488 min)	Cyclododecane	95
		Scan 1842 (14.627 min)	Octadecane	95
		Scan 1981 (15.422 min)	Phenol, 4-(1-methyl-1-phenylethyl)-	97
		Scan 2151 (16.395 min)	Phenol, 4-(1-methyl-1-phenylethyl)-, acetate	97
		Scan 2199 (16.670 min)	Eicosane	97
		Scan 2510 (18.449 min)	Phenol, 4,4'-(1-methylethylidene)bis-	98
		Scan 2522 (18.518 min)	Docosane	97

		Scan 2778 (19.983 min)	9-Octadecenamide, (Z)-	99
		Scan 3221 (22.517 min)	Octocrylene	70

Table S2. The NIST ID of the compounds identified in at least 2 replicates by GCMS in all fungal samples of textile using Amberlite capture resin. Functions and hazards have been compiled from metabolomics databases and PubChem. Compounds identified in the control have been removed.

**Number of compounds found in at least two replicates of *L. edodes***				
**Time Point**	**Compound Name**	**Molecular Formula**	**Function/Activity**	**Hazard Warnings**
**2 Months**	2,4-Di-tert-butylphenol	$C_{14}H_{22}O$	None found	Corrosive, Irritant, Health Hazard, Environmental Hazard
	2-Propenoic acid, tridecyl ester	$C_{16}H_{30}O_2$	None found	Corrosive, Acute Toxic, irritant, Environmental Hazard
	Propanoic acid, 3,3'-thiobis-, didodecyl ester	$C_{30}H_{58}O_4S$	None found	None
	Propanoic acid, 3-mercapto-, dodecyl ester	$C_{15}H_{30}O_2S$	None found	None
**3 Months**	2,4-Di-tert-butylphenol	$C_{14}H_{22}O$	None found	Corrosive, Irritant, Health Hazard, Environmental Hazard
	t-Butylhydroquinone	$C_{10}H_{14}O_2$	2-tert-butylhydroquinone is a member of the class of hydroquinones in which one of the ring hydrogens of hydroquinone is replaced by a tert-butyl group. It has a role as a food antioxidant.	Irritant
	Benzophenone	$C_{13}H_{10}O$	Benzophenone is the simplest member of the class of benzophenones, being formaldehyde in which both hydrogens are replaced by phenyl groups. It has a role as a photosensitizing	Health Hazard

Number of compounds found in at least two replicates of *L. edodes*				
			agent and a plant metabolite.	
	(4-Acetylphenyl)phenylmethane	$C_{15}H_{14}O$	None found	Irritant
	7,9-Di-tert-butyl-1-oxaspiro(4,5)deca-6,9-diene-2,8-dione	$C_{17}H_{24}O_3$	None found	Irritant
	Eicosane	$C_{20}H_{42}$	Alkane oxidation<br><br>Cuticular wax biosynthesis<br><br>alkane biosynthesis	None
	4,4'-Diisopropylbiphenyl	$C_{18}H_{22}$	None found	None
	1-Octadecene	$C_{18}H_{36}$	Terminal olefins biosynthesis	None
	Docosane	$C_{22}H_{46}$	None found	Irritant
	Benzenepropanoic acid, 3,5-bis(1,1-dimethylethyl)-4-hydroxy-, methyl ester	$C_{18}H_{28}O_3$	None found	Irritant, Environmental Hazard
	11-Octadecenoic acid, methyl ester	$C_{19}H_{36}O_2$	None found	Irritant
	Tetracosane	$C_{24}H_{50}$	Cuticular wax biosynthesis	None
	Bis(2-ethylhexyl) phthalate	$C_{23}H_{38}O_4$	Participates in reactions as a substrate	Health Hazard
	Benzenepropanoic acid, 3,5-bis(1,1-dimethylethyl)-4-hydroxy-, octadecyl ester	$C_{35}H_{62}O_3$	None found	Health Hazard, Environmental Hazard
**5 Months**	2,4-Di-tert-butylphenol	$C_{14}H_{22}O$	None found	Corrosive, Irritant, Health Hazard, Environmental Hazard
	Octadecane	$C_{18}H_{38}$	Alkane oxidation<br><br>Cuticular wax biosynthesis<br><br>alkane biosynthesis	Health Hazard
	(4-Acetylphenyl)phenylmethane	$CH_{15}H_{14}O$	None found	Irritant
	Benzenepropanoic acid, 3,5-bis(1,1-dimethylethyl)-4-hydroxy-, methyl ester	$C_{18}H_{28}O_3$	None found	Irritant, Environmental Hazard
	Eicosane	$C_{20}H_{42}$	Alkane oxidation	None

Number of compounds found in at least two replicates of *L. edodes*				
			Cuticular wax biosynthesis<br><br>alkane biosynthesis	
	3,4'-Diisopropylbiphenyl	$C_{18}H_{22}$	None found	None
	Docosane	$C_{22}H_{46}$	None found	Irritant
	Phenol, 2,4-bis(1,1-dimethylethyl)-, phosphite (3:1)	$C_{46}H_{63}O_3P$	None found	None
	Tetracosane	$C_{24}H_{50}$	Cuticular wax biosynthesis	None
	Benzenepropanoic acid, 3,5-bis(1,1-dimethylethyl)-4-hydroxy-, octadecyl ester	$C_{35}H_{62}O_3$	None found	Health Hazard, Environmental Hazard
	Hexacosane	$C_{26}H_{54}$	Cuticular wax biosynthesis	Irritant
**Number of compounds found in at least two replicates of *P. eryngii***				
**Time Point**	**Compound Name**	**Molecular Formula**	**Function/Activity**	**Hazard Warnings**
**2 Months**	2,4-Di-tert-butylphenol	$C_{14}H_{22}O$	None found	Corrosive, Irritant, Health Hazard, Environmental Hazard
	Benzenepropanoic acid, 3,5-bis(1,1-dimethylethyl)-4-hydroxy-, octadecyl ester	$C_{35}H_{62}O_3$	None found	Health Hazard, Environmental Hazard
	Propanoic acid, 3-mercapto-, dodecyl ester	$C_{15}H_{30}O_2S$	None found	None
**3 Months**	2,4-Di-tert-butylphenol	$C_{14}H_{22}O$	None found	Corrosive, Irritant, Health Hazard, Environmental Hazard
	(4-Acetylphenyl)phenylmethane	$CH_{15}H_{14}O$	None found	Irritant
	7,9-Di-tert-butyl-1-oxaspiro(4,5)deca-6,9-diene-2,8-dione	$C_{17}H_{24}O_3$	None found	Irritant
	Benzenepropanoic acid, 3,5-bis(1,1-dimethylethyl)-4-hydroxy-, methyl ester	$C_{18}H_{28}O_3$	None found	Irritant, Environmental Hazard
	Docosane	$C_{22}H_{46}$	None found	Irritant
	Octacosane	$C_{28}H_{58}$	Cuticular wax biosynthesis	None
	Norbolethone	$C_{21}H_{32}O_2$	None found	None

Number of compounds found in at least two replicates of *L. edodes*				
	Propanoic acid, 3-mercapto-, dodecyl ester	$C_{15}H_{30}O_2S$	None found	None
	4,4'-Diisopropylbiphenyl	$C_{18}H_{22}$	None found	None
	5-Octadecene, (E)-	$C_{18}H_{36}$	None found	None
	Benzenepropanoic acid, 3,5-bis(1,1-dimethylethyl)-4-hydroxy-, octadecyl ester	$C_{35}H_{62}O_3$	None found	Health Hazard, Environmental Hazard
**5 Months**	2,4-Di-tert-butylphenol	$C_{14}H_{22}O$	None found	Corrosive, Irritant, Health Hazard, Environmental Hazard
	(4-Acetylphenyl)phenylmethane	$CH_{15}H_{14}O$	None found	Irritant
	1-Ethoxy-2-phenylmethylbenzene	$C_{15}H_{16}O$	None found	None
	Benzenepropanoic acid, 3,5-bis(1,1-dimethylethyl)-4-hydroxy-, methyl ester	$C_{18}H_{28}O_3$	None found	Irritant, Environmental Hazard
	4,4'-Diisopropylbiphenyl	$C_{18}H_{22}$	None found	None
	Benzenepropanoic acid, 3,5-bis(1,1-dimethylethyl)-4-hydroxy-, octadecyl ester	$C_{35}H_{62}O_3$	None found	Health Hazard, Environmental Hazard
	5-Octadecene, (E)-	$C_{18}H_{36}$	None found	None
**Number of compounds found in at least two replicates of *P. ostreatus***				
**Time Point**	**Compound Name**	**Molecular Formula**	**Function/Activity**	**Hazard Warnings**
**2 Months**	2,4-Di-tert-butylphenol	$C_{14}H_{22}O$	None found	Corrosive, Irritant, Health Hazard, Environmental Hazard
	Propanoic acid, 3-mercapto-, dodecyl ester	$C_{15}H_{30}O_2S$	None found	None
	Phenol, 2,4-bis(1,1-dimethylethyl)-, phosphite (3:1)	$C_{46}H_{63}O_3P$	None found	None
	Benzenepropanoic acid, 3,5-bis(1,1-dimethylethyl)-4-hydroxy-, octadecyl ester	$C_{35}H_{62}O_3$	None found	Health Hazard, Environmental Hazard
	Cyclooctane, 1,2-dimethyl-	$C_{10}H_{20}$	None found	None

Number of compounds found in at least two replicates of *L. edodes*				
**3 Months**	2,4-Di-tert-butylphenol	$C_{14}H_{22}O$	None found	Corrosive, Irritant, Health Hazard, Environmental Hazard
	Hexadecanoic acid, methyl ester	$C_{17}H_{34}O_2$	ceramide and sphingolipid recycling and degradation (yeast),<br><br>palmitate biosynthesis<br><br>sphingosine and sphingosine-1-phosphate metabolism,<br><br>stearate biosynthesis I (animals)<br><br>alkane biosynthesis	Irritant
	7,9-Di-tert-butyl-1-oxaspiro(4,5)deca-6,9-diene-2,8-dione	$C_{17}H_{24}O_3$	None found	Irritant
	Eicosane	$C_{20}H_{42}$	Alkane oxidation<br><br>Cuticular wax biosynthesis<br><br>alkane biosynthesis	None
	Propanoic acid, 3-mercapto-, dodecyl ester	$C_{15}H_{30}O_2S$	None found	None
	1-Octadecene	$C_{18}H_{36}$	Terminal olefins biosynthesis	None
	Methyl stearate	$C_{19}H_{38}O_2$	None found	None
	Bromoacetic acid, hexadecyl ester	$C_{18}H_{35}BrO_2$	None found	None
	Tetracosane	$C_{24}H_{50}$	Cuticular wax biosynthesis	None
	Bis(2-ethylhexyl) phthalate	$C_{24}H_{38}O_4$	Participates as a substrate in two known reactions	Health hazard

Number of compounds found in at least two replicates of *L. edodes*				
	Benzenepropanoic acid, 3,5-bis(1,1-dimethylethyl)-4-hydroxy-, octadecyl ester	$C_{35}H_{62}O_3$	None found	Health Hazard, Environmental Hazard
	Norbolethone	$C_{21}H_{32}O_2$	None found	None
	Benzenepropanoic acid, 3,5-bis(1,1-dimethylethyl)-4-hydroxy-, methyl ester	$C_{18}H_{28}O_3$	None found	Irritant, Environmental Hazard
	t-Butylhydroquinone	$C_{10}H_{14}O_2$	2-tert-butylhydroquinone is a member of the class of hydroquinones in which one of the ring hydrogens of hydroquinone is replaced by a tert-butyl group. It has a role as a food antioxidant.	Irritant
**5 Months**	2,4-Di-tert-butylphenol	$C_{14}H_{22}O$	None found	Corrosive, Irritant, Health Hazard, Environmental Hazard
	(4-Acetylphenyl)phenylmethane	$CH_{15}H_{14}O$	None found	Irritant
	Eicosane	$C_{20}H_{42}$	Alkane oxidation<br><br>Cuticular wax biosynthesis<br><br>alkane biosynthesis	None
	1-Octadecene	$C_{18}H_{36}$	Terminal olefins biosynthesis	None
	Docosane	$C_{22}H_{46}$	None found	Irritant
	Phenol, 2,4-bis(1,1-dimethylethyl)-, phosphite (3:1)	$C_{46}H_{63}O_3P$	None found	None
	Benzenepropanoic acid, 3,5-bis(1,1-dimethylethyl)-4-hydroxy-, octadecyl ester	$C_{35}H_{62}O_3$	None found	Health Hazard, Environmental Hazard
	4,4'-Diisopropylbiphenyl	$C_{18}H_{22}$	None found	None

Number of compounds found in at least two replicates of *L. edodes*				
	Tetracosane	$C_{24}H_{50}$	Cuticular wax biosynthesis	None
	Hexacosane	$C_{26}H_{54}$	Cuticular wax biosynthesis	Irritant

Table S3. The NIST ID of the volatile compounds identified in at least 2 replicates by GCMS in all fungal samples of textile using an SPME fibre. Functions and hazards have been compiled from metabolomics databases and PubChem. Derivatives of the same compound are listed together. Compounds identified in the control have been removed.

**Number of compounds found in at least two replicates of *L. edodes***				
**Time point**	**Compound Name**	**Molecular Formula**	**Function/Activity**	**Hazard Warnings**
**3 Months**	Toluene	C_7H_8	Toluene is an important petrochemical. Toluene is used for the synthesis of other aromatic feedstocks and as a solvent.	Flammable, Irritant, Health Hazard
**5 Months**	Pentacosane	$C_{25}H_{52}$	Involved in cuticular wax biosynthesis. It has a role as a semiochemical and a plant metabolite.	Health Hazard
	Tetradecane	$C_{14}H_{30}$	It has a role as a plant metabolite and a volatile oil component	Health Hazard
	Eicosane	$C_{20}H_{42}$	It has a role as a plant metabolite.	None
	Dodecane / Dodecane, 4,6-dimethyl- / Dodecane, 2,6,11-trimethyl- / Dodecane, 1-iodo-	$C_{12}H_{26}$	It has a role as a plant metabolite	Irritant, Health Hazard
	Isolongifolene, 4,5,9,10-dehydro-	$C_{15}H_{20}$	It has a role as an insect attractant, a volatile oil component, an *Aspergillus* metabolite, a human metabolite and a plant metabolite. It is a carbotricyclic compound, a volatile organic compound, a sesquiterpene and a polycyclic olefin.	None
	Toluene	C_7H_8	Toluene is an important petrochemical. Toluene is used for the synthesis of other aromatic feedstocks and as a solvent.	Flammable, Irritant, Health Hazard
	Tetracosane	$C_{24}H_{50}$	Involved in cuticular wax biosynthesis. It has a role as a plant metabolite and a volatile oil component.	None
	Tetradecane, 1-iodo-	$C_{14}H_{29}I$	1-iodotetradecane is an organoiodine. It has a role as a metabolite.	None

**Number of compounds found in at least two replicates of *P. eryngii***				
**Time point**	**Compound Name**	**Molecular Formula**	**Function/Activity**	**Hazard Warnings**
**3 Months**	Dodecane, 1-iodo-	C12H25I	None found	Irritant
	Pentacosane	$C_{25}H_{52}$	Involved in cuticular wax biosynthesis. It has a role as a semiochemical and a plant metabolite.	Health Hazard
	Eicosane	$C_{20}H_{42}$	It has a role as a plant metabolite.	None
	Carbonic acid, eicosyl vinyl ester / Carbonic acid, decyl nonyl ester	CH_2O_3	Nitrogen metabolism	None

	2-(3,4-Dimethoxyphenyl)-2-isopropylpentanedinitrile	$C_{16}H_{20}N_2O_2$	None found	None
**5 Months**	Decane, 2,3,5,8-tetramethyl- / Decane, 2-methyl- / Decane, 3,6-dimethyl- / Decane, 3,7-dimethyl-	$C_{10}H_{22}$	Cuticular wax biosynthesis	Flammable, Health Hazard
	Undecane	$C_{11}H_{24}$	Cuticular wax biosynthesis	Health hazard
	Pentacosane	$C_{25}H_{52}$	Involved in cuticular wax biosynthesis. It has a role as a semiochemical and a plant metabolite.	Health Hazard
	Eicosane	$C_{20}H_{42}$	It has a role as a plant metabolite.	None
	Tetradecane, 1-iodo-	$C_{14}H_{29}I$	1-iodotetradecane is an organoiodine compound. It has a role as a metabolite.	None
	Tetradecane	$C_{14}H_{30}$	It has a role as a plant metabolite and a volatile oil component	Health Hazard
	Benzene, 2,4-diisocyanato-1-methyl-	$CH_3C_6H_3(NCO)_2$	None found	Acute toxic, Health Hazard

**Number of compounds found in at least two replicates of *P. ostreatus***				
**Time point**	**Compound Name**	**Molecular Formula**	**Function/Activity**	**Hazard Warnings**
**3 Months**	Dodecane / Dodecane, 2,6,11-trimethyl-	$C_{12}H_{26}$	It has a role as a plant metabolite	Irritant, Health Hazard
	Eicosane / Eicosane, 1-iodo-	$C_{20}H_{42}$	It has a role as a plant metabolite.	None
	Dimethyl palmitamine	C18H39N	None found	Corrosive, Irritant, Environmental Hazard
**5 Months**	Toluene	C_7H_8	Toluene is an important petrochemical. Toluene is used for the synthesis of other aromatic feedstocks and as a solvent.	Flammable, Irritant, Health Hazard
	Dodecane, 2,6,11-trimethyl- / Dodecane, 4,6-dimethyl- / Dodecane, 1-iodo-	$C_{12}H_{26}$	It has a role as a plant metabolite	Irritant, Health Hazard
	Tetradecane	$C_{14}H_{30}$	It has a role as a plant metabolite and a volatile oil component	Health Hazard
	Eicosane	$C_{20}H_{42}$	It has a role as a plant metabolite.	None
	.alpha.-Corocalene	C15H20	None Found	None
	Toluene	C_7H_8	Toluene is an important petrochemical. Toluene is used for the synthesis of other aromatic feedstocks and as a solvent.	Flammable, Irritant, Health Hazard
